# Transcriptomic Analysis of Venom Secretion in *Achelura yunnanensis*: Lipid Metabolism, Redox Reactions, and Structural Adaptations

**DOI:** 10.3390/insects16060588

**Published:** 2025-06-03

**Authors:** Ping Liu, Hui-Qin Zhu, Si-Ming Wang, Yu-Qian Wang, Zhen-Yuan Ruan, Lu Qiao, Xing-Xing Wu, Qing-Hua Yan, Ya-Ping Lu, Bing Bai, Wei-Feng Ding

**Affiliations:** 1 College of Forestry, Jindian Campus, Yunnan Forestry Technological College, Kunming 650225, China; liuping0101@aliyun.com (P.L.); zhq10302025@163.com (H.-Q.Z.); wsm18707@163.com (S.-M.W.); yuxianwang92@gmail.com (Y.-Q.W.); edith0727@126.com (Z.-Y.R.); qiaoqiaotantan@163.com (L.Q.); wuxingxing9906@163.com (X.-X.W.); 15087011600@126.com (Q.-H.Y.); luyaping0312@163.com (Y.-P.L.); 2 Institute of Highland Forest Science, Chinese Academy of Forestry, Kunming 650224, China; 3 Yunnan Key Laboratory of Breeding and Utilization of Resource Insects, Kunming 650224, China

**Keywords:** insect venom secretion, transcriptomics, lipid metabolism, redox reactions, surface protective structures, resource reallocation, *Achelura yunnanensis*, defensive mechanisms, pest control

## Abstract

As a key pest damaging urban greenery in Yunnan, China, *Achelura yunnanensis* larvae secrete venom for defense, yet the molecular basis of this process remains poorly understood. This study aimed to uncover the molecular mechanisms of venom secretion by comparing the dorsal epidermis tissue (LDET) with the larval proleg tissue (LP). We performed transcriptomic analysis using RNA sequencing to identify differentially expressed genes between LDET and LP (10 biological replicates per tissue type), followed by functional enrichment and gene expression correlation analyses to explore tissue-specific characteristics. LDET exhibited significant upregulation of pathways related to lipid metabolism, redox reactions, and surface protective structure formation, suggesting their roles in venom stabilization, activation, and safe secretion. Conversely, genes linked to non-venom-related functions, such as extracellular matrix organization and epidermal development, were downregulated in LDET, indicating resource reallocation toward venom production. These findings reveal a multi-component mechanism in LDET that supports venom secretion through metabolic and structural adaptations, with lipid metabolism genes constituting 18.3% of total differentially expressed genes, highlighting evolutionary trade-offs in insect defense. This study provides new insights into insect venom secretion and offers potential targets for pest control strategies.

## Simple Summary

Venom in insects, like the larvae of *Achelura yunnanensis*, helps them defend against predators, but how they produce and release this venom is not fully understood. In this study, we explored the differences between two parts of the larvae: the dorsal epidermis tissue, which makes venom, and the larval proleg tissue, which does not. We looked at the activity of thousands of genes in these tissues to understand what makes the venom-producing tissue special. Our findings show that the venom tissue relies heavily on processes that create fats, manage chemical reactions, and build protective layers on its surface. These processes help the tissue store and release venom safely while protecting itself from harm. This work reveals the complex ways insects prepare their defenses, which could help us develop new methods to control pest insects that harm crops or spread diseases. By understanding how insects like these larvae protect themselves, we can find better ways to manage them in agriculture and improve safety for farmers and the environment.

## 1. Introduction

Insect chemical defenses represent a sophisticated interplay of evolutionary adaptations and ecological interactions. Among these, *Achelura yunnanensis*, a Lepidopteran species in the *Zygaenidae* family, stands out for its larvae’s ability to secrete venom from dorsal epidermis tissue (LDET) as a defense against predators such as ants, birds, and mammals [1,2]. Prevalent in Yunnan, China, these larvae feed primarily on *Rosaceae* plants like *Prunus cerasoides*, causing significant damage to urban greenery and posing challenges to ecological management [1,3]. The venom, characterized by cyanogenic glycosides linamarin and lotaustralin, is released via specialized hair tufts and glands, offering a potent deterrent [1,2]. However, the precise mechanisms of cyanogenic glycoside synthesis and their contribution to venom secretion in *A. yunnanensis* remain unclear, prompting further investigation into the molecular basis of this defense strategy.

Research on *Zygaenidae* species has traditionally focused on two primary mechanisms for cyanogenic glycoside acquisition: de novo biosynthesis or sequestration from host plants [4,5]. Isotope-labeling studies in related species, such as *Zygaena trifolii*, suggest that these compounds can be synthesized in tissues like hemolymph and defensive glands or accumulated from cyanogenic diets [6,7]. Similarly, *Nymphalidae* insects demonstrate the ability to uptake and modify plant-derived glycosides for defense [5,6]. In *A. yunnanensis*, bioactivity assays confirm linamarin’s antifeedant effects against *Tapinoma melanocephalum* [2], yet the molecular machinery enabling the secretion of these cyanogenic glycosides remains unexplored.

To address this gap, we conducted a transcriptomic analysis comparing LDET with larval proleg tissue (LP) as a control. We hypothesized that venom secretion in LDET involves not only cyanogenic glycoside production but also a broader network of metabolic and structural processes. Our findings reveal an unexpected profile: rather than a dominant signature of glycoside biosynthesis, LDET exhibits the significant upregulation of genes related to lipid metabolism, redox reactions, and surface protective structures. These results suggest a complex, multi-component mechanism supporting venom function, offering new insights into the diversity of insect chemical defenses and potential targets for eco-friendly pest control strategies against *A. yunnanensis*.

## 2. Materials and Methods

### 2.1. Insect Collection and Sample Preparation

**Figure 1:**
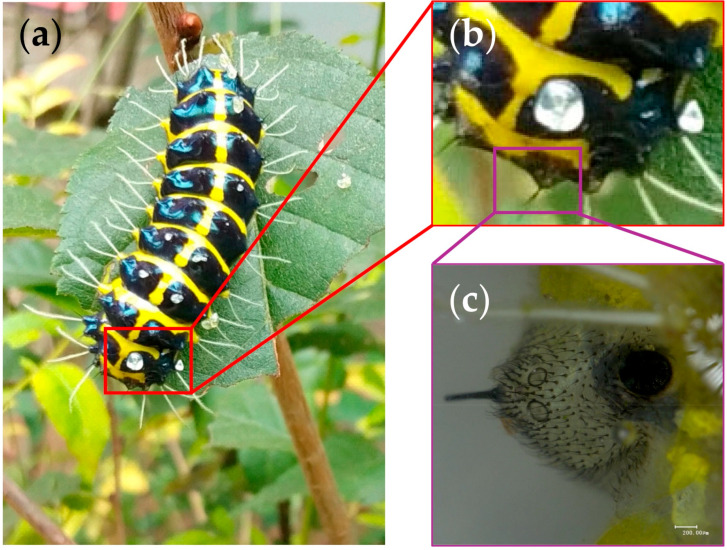
Morphology and venom hair microstructure of *Achelura yunnanensis* larvae. (**a**) Overall larval morphology, showing alternating black and yellow segments with white setae along the body sides. (**b**) Close-up of venom hairs on the larval dorsum, with transparent venom droplets at seta tips; purple box indicates the sampling area for (**c**). (**c**) Photomicrograph of subcutaneous tissue at the venom hair base (LDET), revealing dense microstructures associated with venom secretion. Scale bar: 200 μm.

Larvae of *A. yunnanensis* were collected from *Prunus cerasoides* plants near Jindian Park, Kunming, Yunnan Province, China, in June 2024. To ensure sample purity, larvae were surface-sterilized with 70% ethanol and immediately dissected under a stereomicroscope following cold anesthesia (4 °C) to prevent stress-induced changes in gene expression. Dorsal epidermis tissue (LDET), identified as the venom-secreting region due to its specialized hair tufts and glandular structures [1], and larval proleg tissue (LP), selected as a non-secretory control due to its distinct locomotory function and developmental comparability to LDET, were carefully isolated (see Figure 1 for morphology and sampling details). The choice of LP as a control facilitates the isolation of venom-specific molecular signatures by providing a contrast with the venom-secreting function of LDET. Ten biological replicates per tissue type (LDET01–LDET10 and LP01–LP10) were prepared, flash-frozen in liquid nitrogen, and stored at −80 °C until RNA extraction.

### 2.2. RNA Extraction and Sequencing

Total RNA was extracted from LDET and LP samples using TRIzol reagent (Invitrogen, Waltham, MA, USA, Cat#15596026). RNA concentration and purity were assessed with a NanoDrop 2000 spectrophotometer (Thermo Scientific, Waltham, MA, USA), and integrity was confirmed via agarose gel electrophoresis and an Agilent 2100 Bioanalyzer (Agilent, Santa Clara, CA, USA). cDNA libraries were constructed using the TruSeq RNA Library Prep Kit (Illumina, San Diego, CA, USA, Cat#RS-122-2001). Libraries were quantified initially with Qubit 2.0 (Invitrogen, Waltham, MA, USA), followed by insert size validation using the Agilent 2100 Bioanalyzer (Agilent Technologies, Inc., Santa Clara, CA, USA), and final quantification via qRT-PCR. Sequencing was performed on the Illumina NovaSeq X Plus platform (Illumina, San Diego, CA, USA), generating paired-end reads.

### 2.3. Transcriptome Assembly and Annotation

Raw reads were filtered using Fastp (v0.23.2) [8] to remove adapters and low-quality sequences, producing clean reads for analysis. De novo assembly was conducted with Trinity (v2.15.1) [9], with quality assessed using TransRate (v1.0.3) [10] and BUSCO (v5.7.1) [11]. Assembled transcripts were annotated against NR, Swiss-Prot, KEGG, GO, Pfam, and EggNOG databases using BLAST (v2.12.0). Gene expression levels (TPM) were quantified with RSEM (v1.3.3) [12].

### 2.4. Differential Expression and Functional Analysis

Differential gene expression between LDET and LP was analyzed using DESeq2 (v1.44.0) [13], applying a threshold of |log2FC| ≥ 1 and an adjusted *p*-value < 0.05. DESeq2 analysis was performed with independent filtering (alpha = 0.05) to optimize the detection of differentially expressed genes. The functional enrichment of differentially expressed genes (DEGs) was performed using GO and KEGG databases. Principal component analysis (PCA), Venn diagrams, and volcano plots were generated on the MajiBio Cloud platform (v1.0) [14]. Violin plots were created using the R (v4.4.2) package ggplot2 (v3.5.1), while additional visualizations, including ridgeline plots and heatmaps, were created using TBtools-II (v2.147) [15] and the R (v4.4.2) package ggridges (v0.5.6).

### 2.5. Validation of RNA-Seq Data by qRT-PCR

To validate the RNA-seq results, quantitative real-time PCR (qRT-PCR) was performed on ten differentially expressed genes (see Table S11 for gene descriptions) selected from lipid metabolism, redox reaction, wax biosynthesis, and extracellular matrix-related pathways. The elongation factor 1-alpha-like gene (TRINITY_DN22741_c0_g1) was used as the reference gene due to its stable expression across samples. Total RNA from ten samples was reverse transcribed using the TSINGKE Goldenstar RT6 cDNA Synthesis Kit (Tsingke Biotechnology Co., Ltd., Beijing, China, Cat# TSK301S). qRT-PCR was conducted using TSINGKE 2×T5 Fast qPCR Mix (SYBR Green I) (Tsingke Biotechnology Co., Ltd., Cat# TSE202) on a QuantStudio 1 Plus Real-Time PCR System (Applied Biosystems, Waltham, MA, USA, Cat# A51685). Each 20 μL reaction contained 1 μL of 5 ng/μL cDNA, 10 μL qPCR Mix (SYBR Green I), and 0.5 μM each of the forward and reverse primers. Gene-specific primers, designed and synthesized by Beijing Tsingke Biotechnology Co., Ltd., are listed in Table S11. The qRT-PCR program consisted of pre-denaturation at 95 °C for 2 min, followed by 40 cycles of 95 °C for 15 s, 60 °C for 20 s, and 72 °C for 20 s, with a final annealing step at 95 °C for 5 s and 65 °C for 1 min. Ct values were normalized to the reference gene using the 2^−ΔΔCt^ method, and statistical significance was assessed via *t*-tests. Primer sequences and validation results are provided in Supplementary Table S11.

### 2.6. Gene Correlation and Network Analysis

Spearman correlation analysis was conducted on TPM values using R (v4.4.2), identifying co-expressed genes with |ρ| ≥ 0.5 and q-value ≤ 0.05 (adjusted via Benjamini–Hochberg). Correlation networks were constructed and visualized in Cytoscape (v3.10.3) [16], with node centrality calculated using the CytoNCA plugin (v2.1.6) [17] to identify key genes.

## 3. Results

### 3.1. Distinct Transcriptomic Profiles of Venom-Secreting Tissue

To investigate the molecular basis of venom secretion in *Achelura yunnanensis* larvae, we performed high-throughput RNA sequencing on dorsal epidermis tissue (LDET), the venom-secreting region, and proleg tissue (LP) as a non-secretory control, using the Illumina NovaSeq X Plus platform. Sequencing generated 886 million reads totaling 877.04 Gb of raw data, with over 95% of bases achieving a Q30 score, indicating a high-quality output (Table S1 in Supplementary Materials). De novo assembly with Trinity yielded 22,028 unigenes and 32,856 transcripts, with N50 lengths of 2632 bp and 2738 bp, respectively, and BUSCO completeness scores of 96.2% (unigenes) and 97.3% (transcripts) (Table S2). These metrics confirm the reliability of the transcriptome for downstream analysis.

**Figure 2:**
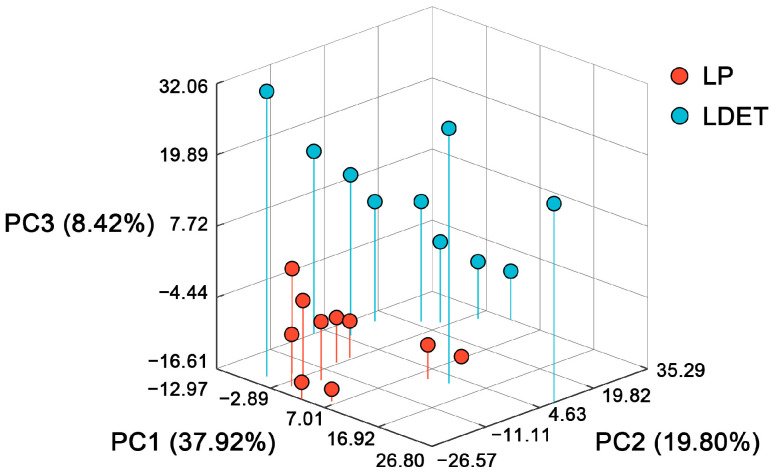
Principal component analysis (PCA) of transcriptomic profiles in *Achelura yunnanensis* larvae. A 3D scatter plot illustrating the distribution of LDET (red dots, *n* = 10) and LP (blue dots, *n* = 10) samples based on gene expression data. The *x*-axis represents PC1 (37.92% variance), the *y*-axis PC2 (19.80% variance), and the *z*-axis PC3 (8.42% variance). LDET samples cluster in the lower-left quadrant, while LP samples occupy the upper-right quadrant, highlighting distinct transcriptomic identities between venom-secreting and non-secretory tissues.

Principal component analysis (PCA) revealed distinct transcriptomic profiles between LDET and LP (Figure 2). The first three principal components (PC1: 37.92%, PC2: 19.80%, PC3: 8.42%) accounted for 66.14% of the total variance (Table S3), effectively separating LDET samples (*n* = 10, red dots) in the lower-left quadrant from LP samples (*n* = 10, blue dots) in the upper-right quadrant. This clear clustering underscores the specialized role of LDET in venom secretion, distinguishing it from the non-secretory LP tissue and setting the stage for identifying venom-related molecular signatures.

### 3.2. Unexpected Absence of Cyanogenic Glycoside Biosynthesis Signature

**Figure 3:**
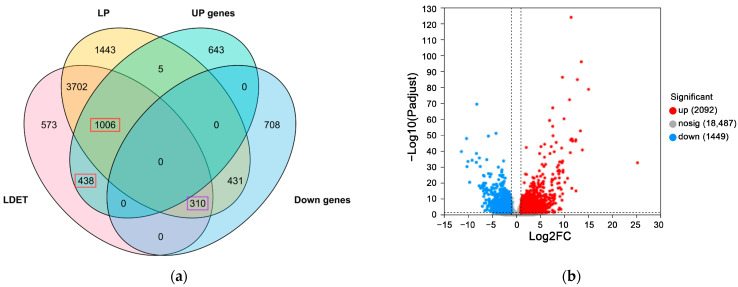
Differential gene expression patterns between LDET and LP tissues in *Achelura yunnanensis* Larvae. (**a**) Venn diagram depicting gene expression overlap between LDET (*n* = 10) and LP (*n* = 10) samples with expression levels > 1. The left circle (LDET) includes 6029 genes, with 1011 unique to LDET and 5018 shared with LP. The right circle (LP) includes 6897 genes, with 1879 unique to LP. Red boxes highlight significantly upregulated genes in LDET (438 unique, 1006 shared), and the purple box denotes 310 shared downregulated genes (threshold: |log2FC| ≥ 1, padjust < 0.05). (**b**) Volcano plot illustrating the distribution of differentially expressed genes between LDET (*n* = 10) and LP (*n* = 10). The *x*-axis represents log2 fold change (log2FC), and the *y*-axis represents −log10 adjusted *p*-value. Red dots indicate genes significantly upregulated in LDET (1444 total), blue dots denote genes upregulated in LP (downregulated in LDET), and gray dots represent non-significant genes (threshold: |log2FC| ≥ 1, padjust < 0.05).

To identify molecular signatures associated with venom secretion, we conducted differential gene expression analysis between LDET and LP tissues using DESeq2, applying a threshold of |log2FC| ≥ 1 and adjusted *p*-value < 0.05. A total of 1444 genes were significantly upregulated in LDET, including 438 unique to LDET and 1,006 shared with LP but more highly expressed in LDET, while 310 genes were significantly downregulated in both tissues (Figure 3a; Table S4). The volcano plot further illustrates this distribution, with red dots marking LDET-upregulated genes and blue dots indicating LP-upregulated (LDET-downregulated) genes (Figure 3b). Notably, among the 5018 genes expressed in both tissues (expression level > 1), 1011 were unique to LDET and 1879 to LP (Figure 3a; Table S5).

Contrary to expectations, genes directly linked to cyanogenic glycoside biosynthesis, such as those in the CYP and UGT families (e.g., homologs of *Zygaena*’s CYP405A2, CYP332A3, and UGT33A1), did not exhibit significant upregulation in LDET (Table 1). The differential expression analysis of five candidate genes revealed that none met the significance threshold (Padj < 0.05), with log2FC values (LDET vs. LP) ranging from −0.971 to 2.606 (Table 1). Notably, *TRINITY_DN1005_c0_g1* (*CYP405A2*) showed higher expression in LP (mean TPM = 470.398) compared to LDET (mean TPM = 213.097), with a log2FC of 1.212 and Padj of 0.7321, indicating no significant upregulation in LDET. Similarly, *TRINITY_DN7282_c0_g3* (*UGT33A1*) was slightly downregulated in LDET (log2FC = 0.971, Padj = 0.9756). This absence challenges the conventional assumption that venom secretion in *Zygaenidae* larvae primarily relies on the de novo synthesis of cyanogenic glycosides, as observed in species like *Zygaena trifolii* [6,7]. Instead, the differential expression profile suggests that LDET employs alternative mechanisms to support venom function, prompting the further exploration of metabolic and structural pathways overrepresented in the upregulated gene set.

### 3.3. Lipid Metabolism and Redox Reactions as Pillars of Venom Function

**Figure 4:**
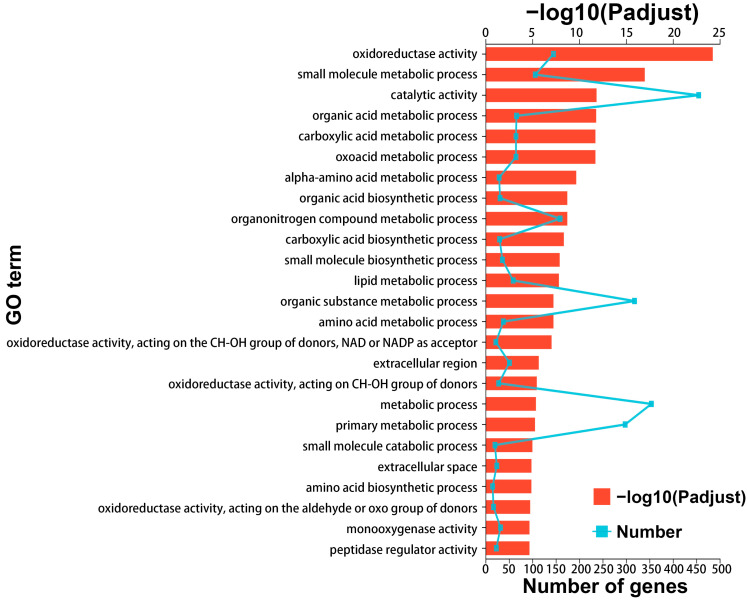
GO enrichment analysis of upregulated genes in lDET. GO enrichment analysis displaying the top 25 enriched GO terms for 1444 genes significantly upregulated in LDET compared to LP (threshold: |log2FC| ≥ 1, Padjust < 0.05). The plot combines bars and a line, with the top *x*-axis representing −log10(Padjust) values (red bars, indicating enrichment significance) and the bottom *x*-axis showing the number of genes (light blue line). The *y*-axis lists GO terms, including oxidoreductase activity, metabolic processes, and catalytic activity.

**Figure 5:**
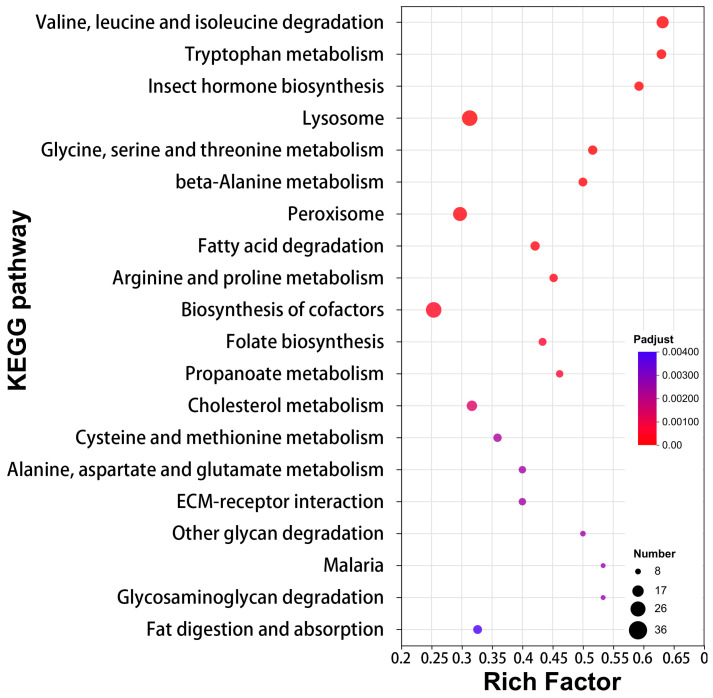
KEGG pathway enrichment analysis of upregulated genes in LDET. KEGG pathway enrichment analysis illustrating the top 25 enriched pathways for genes upregulated in LDET. The *x*-axis represents the Rich Factor; the *y*-axis lists full pathway names.

To elucidate the molecular mechanisms supporting venom function in LDET, we performed functional enrichment analysis on the 1444 genes significantly upregulated in LDET compared to LP. Gene Ontology (GO) analysis revealed that these genes were predominantly enriched in catalytic activity (GO:0003824, 455 genes, Padjust = 1.42 × 10^−12^), oxidoreductase activity (GO:0016491, 145 genes, Padjust = 5.56 × 10^−25^), small molecule metabolic process (GO:0044281, 106 genes, Padjust = 1.01 × 10^−17^), and organic acid metabolic process (GO:0006082, 66 genes, Padjust = 1.56 × 10^−12^), alongside other terms like lipid metabolic process (GO:0006629, 59 genes, Padjust = 1.48 × 10^−8^) (Figure 4; detailed results in Table S6). Similarly, KEGG pathway analysis highlighted significant enrichment in amino acid metabolism pathways, such as “Valine, leucine and isoleucine degradation” (map00280, 24 genes, Padjust = 5.06 × 10^−11^) and “Tryptophan metabolism” (map00380, 17 genes, Padjust = 1.03 × 10^−7^), as well as pathways related to redox reactions and cellular processes, including “Peroxisome” (map04146, 30 genes, Padjust = 8.57 × 10^−5^) and “Lysosome” (map04142, 36 genes, Padjust = 3.23 × 10^−6^) (Figure 5; detailed results in Table S7). Additionally, lipid metabolism pathways like “Fatty acid degradation” (map00071, 16 genes, Padjust = 0.00013511) were enriched, supporting the role of lipid metabolism in venom production. Notably, lipid metabolism genes (GO:0006629, 59 genes) constituted 4.1% of the total 1444 DEGs, underscoring their importance in venom function.

**Figure 6:**
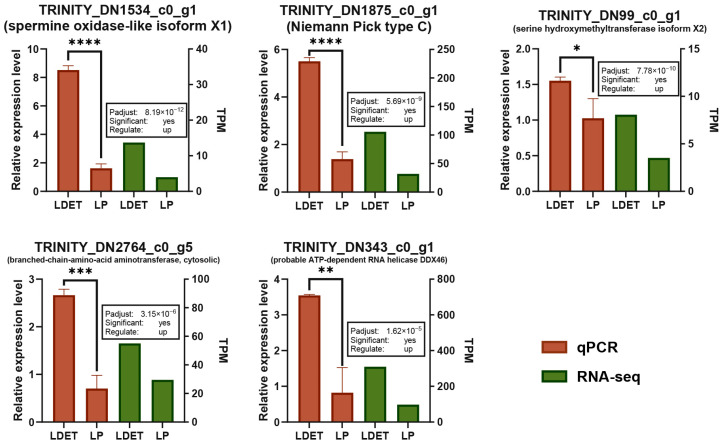
Validation of expression profiles of key interacting genes in LDET tissue. Validation of expression levels for five genes significantly upregulated in LDET compared to LP (Padjust < 0.05, DESeq2 analysis). Each gene is represented by a bar plot, with red bars on the left showing qPCR relative expression levels and green bars on the right showing RNA-seq data (TPM). A text box above green plot indicates Padjust values, significance (yes/no), and regulation direction (up/down). Significance levels marked by * (*p* < 0.05), ** (*p* < 0.01), *** (*p* < 0.001), and **** (*p* < 0.0001). The *x*-axis represents sample groups (LDET and LP).

To further explore the interactions among upregulated genes, we constructed an expression correlation network for the 1444 upregulated genes and ranked them by their degree of connectivity (Degree). The top 50 genes with the highest degree were selected as key interacting genes. A bar plot demonstrated the significant upregulation of five representative genes in LDET samples compared to LP (Padj < 0.05, DESeq2 analysis; Figure 6; detailed correlation and centrality analyses in Table S8, qRT-PCR validation results in Table S11). Notable examples include *Niemann-Pick type C protein* (*TRINITY_DN1875_c0_g1*, involved in cholesterol transport), which supports lipid metabolism and venom stability, and *spermine oxidase-like isoform X1* (*TRINITY_DN1534_c0_g1*, associated with redox reactions), aligning with the enriched oxidoreductase activity in GO analysis (Figure 4). Additionally, genes like *branched-chain-amino-acid aminotransferase*, *cytosolic* (*TRINITY_DN2764_c0_g5*), and *serine hydroxymethyltransferase isoform X2* (*TRINITY_DN99_c0_g1*) are linked to amino acid metabolism, consistent with KEGG pathways such as “Valine, leucine and isoleucine degradation” and “Glycine, serine and threonine metabolism” (Figure 5). The probable ATP-dependent RNA helicase DDX46 (*TRINITY_DN343_c0_g1*) suggests a role in RNA processing, potentially contributing to the post-translational modification of venom proteins. These findings highlight a coordinated network where lipid metabolism, redox reactions, and amino acid metabolism collectively support venom function in LDET.

**Figure 7:**
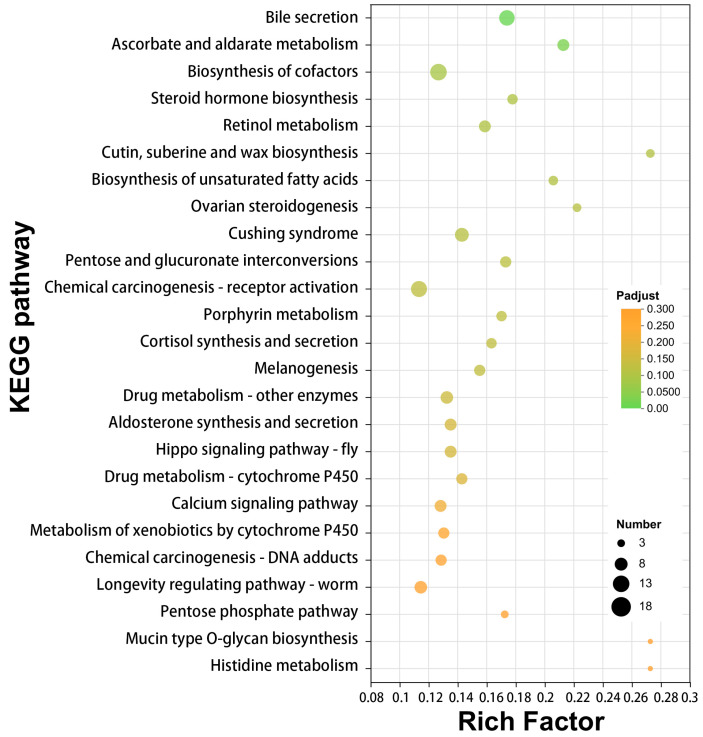
KEGG pathway enrichment analysis of downregulated genes in LDET. KEGG pathway enrichment analysis showing the top 25 enriched pathways for genes significantly downregulated in LDET compared to LP. The *x*-axis represents the Rich Factor; the *y*-axis lists full pathway names, with the bubble size indicating gene count and color intensity reflecting Padjust values.

Conversely, the KEGG pathway analysis of downregulated genes in LDET revealed enrichment in metabolic and endocrine-related pathways, such as “Bile secretion” (map04976, 16 genes, Padjust = 0.019502159) and “Steroid hormone biosynthesis” (map00140, 8 genes, Padjust = 0.112536941) (Figure 7; detailed results in Table S9). This downregulation suggests that LDET may reduce certain metabolic and endocrine activities, potentially redirecting resources toward venom-specific functions. Together, these data point to a coordinated metabolic framework where lipid metabolism facilitates venom storage and stability, while redox reactions may activate or modify venom components for effective defense.

### 3.4. Structural Adaptations Supporting Venom Secretion

**Figure 8:**
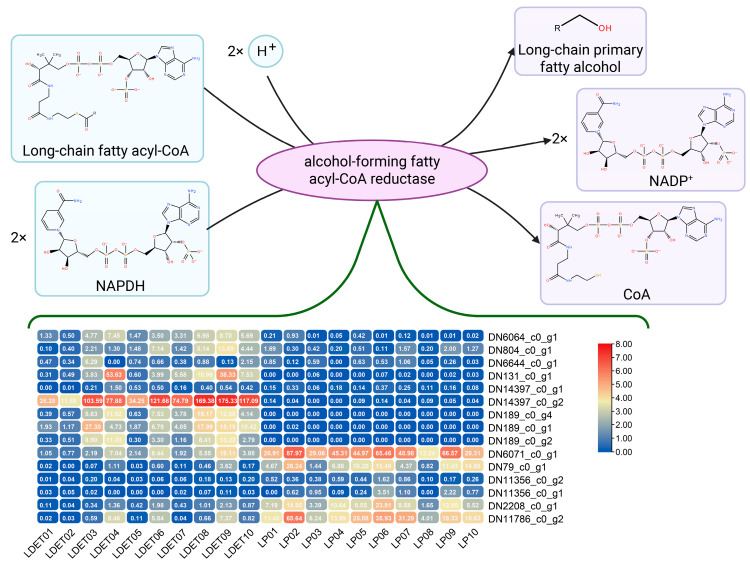
Expression heatmap of *FAR* and downregulated genes in the “Cutin, Suberin and Wax Biosynthesis” pathway. A heatmap embedded within the “Cutin, suberin and wax biosynthesis” pathway (map00073), showing the expression levels (TPM) of 15 genes in LDET (*n* = 10) and LP (*n* = 10) samples. The pathway step catalyzed by alcohol-forming fatty acyl-CoA reductase (*FAR*) (conversion of long-chain fatty acyl-CoA to long-chain primary fatty alcohol) is highlighted. Expression levels are indicated by a blue-to-red color gradient (blue: 0 TPM, red: 8 TPM). FAR genes (black labels, e.g., TRINITY_DN6064_c0_g1, *TRINITY_DN804_c0_g1*) are significantly upregulated in LDET, supporting wax biosynthesis, while downregulated genes (green labels, e.g., TRINITY_DN6071_c0_g1, *TRINITY_DN79_c0_g1*) indicate a reallocation of resources from non-venom-related functions.

Venom secretion in LDET is not only supported by metabolic reprogramming but also by structural adaptations that facilitate the production, storage, and delivery of venom components. To investigate these adaptations, we analyzed the KEGG pathway enrichment of upregulated genes in LDET, focusing on pathways related to structural protection. The “Cutin, suberin and wax biosynthesis” pathway (map00073) was significantly enriched among the upregulated genes (9 genes), suggesting that LDET enhances the production of protective surface structures, such as wax layers, to shield venom-secreting tissues from environmental stress or self-toxicity. Notably, the alcohol-forming fatty acyl-CoA reductase (*FAR*) genes, which catalyze the conversion of long-chain fatty acyl-CoA to long-chain primary fatty alcohol—a critical step in wax biosynthesis—were significantly upregulated in LDET, showing an average 4.6-fold increase (Log2FC = 2.2 ± 0.4, *t*-test, *p* = 0.002). A heatmap of 15 genes, including six downregulated genes, revealed distinct expression patterns between LDET and LP (Figure 8). This upregulation of FAR genes likely contributes to a hydrophobic barrier that stabilizes venom components during secretion, while the downregulation of other genes may reduce the maintenance of non-essential structures, such as general extracellular matrix (ECM) components. Additionally, pathways related to ECM remodeling, such as “ECM–receptor interaction” (map04512, 3 genes), were enriched among upregulated genes, suggesting that LDET may undergo structural modifications to facilitate venom delivery, possibly by enhancing the extracellular space for venom transport.

**Figure 9:**
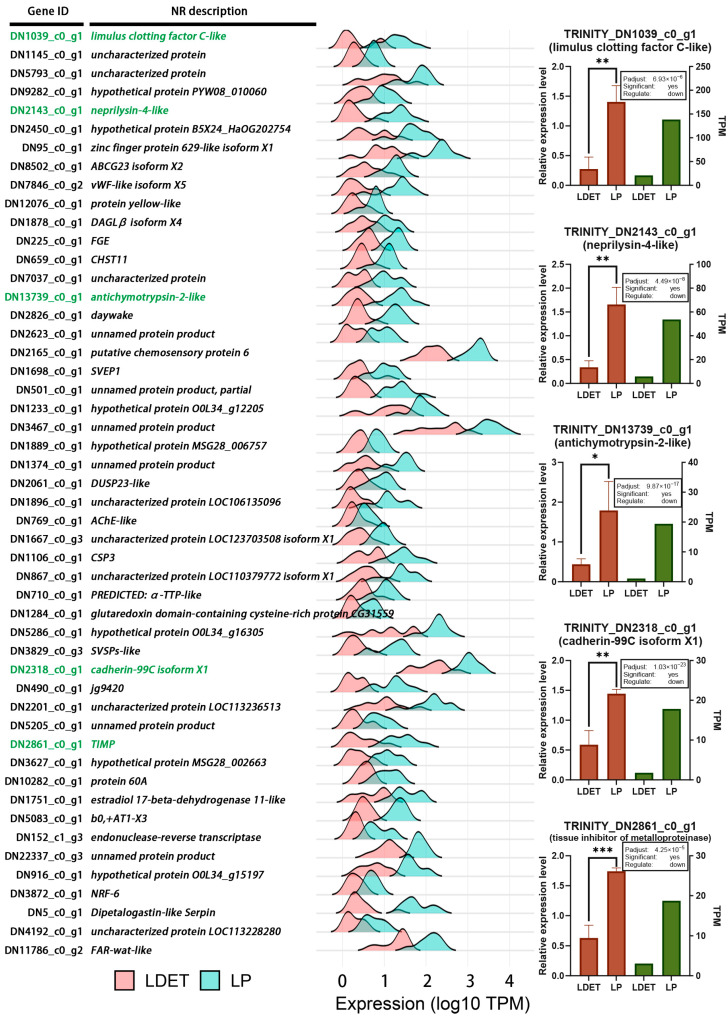
Expression profiles of downregulated genes in LDET tissue. A ridgeline plot displaying the expression levels (log10(TPM)) of 50 genes significantly downregulated in LDET (selected based on expression correlation analysis, degree top 50, log2FC < −1, Padjust < 0.05) across LDET (*n* = 10, pink) and LP (*n* = 10, light blue) samples. The *x*-axis shows log10(TPM) values, and the left side lists gene IDs and NR descriptions (e.g., limulus clotting factor C-like), demonstrating consistent downregulation in LDET. The right compares qPCR and RNA-seq data with bar plots. qPCR results are shown in red bars, RNA-seq results in green bars, with a text box above indicating Padjust values, significance (yes/no), and regulation direction (up/down). Significance levels are marked by * (*p* < 0.05), ** (*p* < 0.01), and *** (*p* < 0.001). The *x*-axis represents sample groups (LDET and LP), and the *y*-axis shows relative expression levels or TPM values.

To further explore the structural adaptations, we examined the expression profiles of downregulated genes in LDET, which may reflect a reallocation of resources away from non-venom-related structural functions. A ridgeline plot of 50 downregulated genes (selected based on expression correlation analysis, degree top 50, log2FC < −1, Padjust < 0.05) revealed consistent suppression in LDET compared to LP (Figure 9, Table S10). Expression levels were log10-transformed to better visualize differences across a wide range of TPM values. Notably, genes associated with ECM organization and structural integrity, such as *TRINITY_DN1039_c0_g1* (*limulus clotting factor C-like*), *TRINITY_DN2143_c0_g1* (*neprilysin-4-like*), *TRINITY_DN13739_c0_g1* (*antichymotrypsin-2-like*), *TRINITY_DN2318_c0_g1* (*cadherin-99C isoform X1*), and *TRINITY_DN2861_c0_g1* (*tissue inhibitor of metalloproteinase*), were significantly downregulated in LDET. Furthermore, qRT-PCR validation confirmed the suppression of these five genes in LDET (Figure 9, Table S11), supporting the consistency of RNA-seq results. This suppression suggests that LDET may reduce the maintenance of general ECM structures and cell adhesion processes, redirecting resources toward venom-specific structural adaptations, such as wax biosynthesis and ECM remodeling for venom delivery.

Together, these findings suggest a multi-component mechanism where metabolic pathways, such as lipid metabolism, support structural adaptations in LDET. The upregulation of “Cutin, suberine and wax biosynthesis”, particularly through *FAR* genes, likely provides a protective barrier for venom-secreting tissues, while ECM remodeling facilitates venom transport and secretion. Conversely, the downregulation of ECM-related and other non-venom-related genes may optimize resource allocation for venom-specific functions. This “metabolism–structure synergy” model underscores the intricate balance between metabolic reprogramming and structural adaptations in LDET, ensuring efficient venom production and delivery for defense.

## 4. Discussion

This study provides novel insights into the molecular mechanisms underlying venom secretion in the dorsal epidermis tissue (LDET) of *A. yunnanensis* larvae by comparing its transcriptomic profile with that of larval proleg tissue (LP). Our initial hypothesis aimed to identify genes involved in cyanogenic glycoside biosynthesis, given their role as defensive compounds in *A. yunnanensis* [1,2]. However, the lack of significant enrichment in this pathway in LDET suggests an evolutionary shift, where *A. yunnanensis* likely sequesters these compounds from its host plant, *P. cerasoides*, rather than relying on energetically costly de novo synthesis. This adaptation may reflect an ecological strategy to redirect metabolic resources toward venom stability and secretion mechanisms, such as lipid metabolism and structural adaptations, which emerged as dominant processes in our transcriptomic analysis.

Our findings suggest that LDET employs a multi-faceted strategy involving lipid metabolism, redox reactions, and the formation of surface protective structures to facilitate venom secretion and maintain tissue integrity. This lipid–redox–structure triad resembles mechanisms observed in snake venom gland systems, where lipid metabolism supports venom stability, redox reactions facilitate toxin activation, and structural adaptations ensure safe secretion [18], suggesting convergent evolution in venom secretion strategies across distant taxa. The immediate dissection of samples preserved the natural gene expression profiles, providing a reliable basis for identifying these mechanisms without the confounding effects of external variables such as starvation.

One of the most striking observations from our analysis is the prominent role of lipid metabolism in LDET. The absence of significant upregulation in cyanogenic glycoside biosynthesis genes may indicate that *A. yunnanensis* has evolved a dependency on exogenous cyanogenic glycosides from *P. cerasoides*, a strategy that conserves energy for other critical functions. This shift could explain the prominence of lipid metabolism in LDET, which likely serves to stabilize venom components, such as cyanogenic glycosides or their derivatives, within lipid complexes. Such complexes may enhance solubility and prevent premature hydrolysis, a process that could be advantageous under natural feeding conditions. Furthermore, the upregulation of cholesterol transport-related genes suggests a role in reinforcing membrane integrity, potentially mitigating oxidative stress from residual cyanogenic compounds or their breakdown products, aligning with an adaptive response to environmental variability. In many insects, toxic secondary metabolites are stored in lipid complexes to maintain stability across developmental stages [19]. The enhanced lipid metabolism in LDET may support the formation of such complexes, ensuring that venom components remain stable during storage and secretion. Additionally, the involvement of lipid-related pathways in antioxidant protection suggests a mechanism to prevent oxidative degradation of venom components, a critical factor for maintaining their potency [20].

Another key aspect of LDET’s venom secretion mechanism is the active redox reactions, which may be linked to the activation or detoxification of venom components. Cyanogenic glycosides, while relatively non-toxic in their native form, often require enzymatic hydrolysis or oxidative activation to release hydrogen cyanide, a potent defensive compound [21]. The enrichment of redox-related pathways in LDET, particularly those involving oxidoreductases, suggests a sophisticated mechanism for modulating venom toxicity. These pathways may catalyze the oxidative activation of sequestered cyanogenic glycosides or their derivatives into hydrogen cyanide, a process potentially triggered by environmental cues such as mechanical damage. This activation could involve electron transfer cascades that enhance the efficiency of toxin release, while simultaneously generating reactive oxygen species (ROS) that require tight regulation to prevent cellular damage. The upregulation of redox enzymes may thus reflect an evolutionary adaptation to balance toxin potency with tissue protection, a dual role that mirrors the redox homeostasis observed in venom glands of higher organisms like snakes [18].

The formation of surface-protective structures in LDET represents a structural adaptation that likely enhances venom storage and secretion while preventing self-harm. The biosynthesis of wax layers, potentially driven by upregulated fatty acid metabolism, could involve the deposition of long-chain fatty alcohols, which form a protective cuticle that minimizes autotoxicity. This adaptation may also facilitate venom delivery by establishing extracellular reservoirs, a process that could involve dynamic remodeling of the ECM to accommodate secretion pressure, reflecting an evolutionary trade-off between structural integrity and defensive efficiency in *A. yunnanensis*. Similar structural adaptations have been reported in other Lepidopteran insects, where defensive glands are often lined with protective coatings to isolate toxic secretions [22].

Gene expression correlation analysis further revealed a coordinated regulatory network in LDET, suggesting that venom secretion is tightly regulated at multiple levels. The downregulation of genes associated with physiological regulation, chemosensation, and epidermal development in LDET suggests an evolutionarily refined resource reallocation strategy [23]. This suppression likely reflects a trade-off where *A. yunnanensis* prioritizes venom production over non-essential functions, driven by selective pressure to enhance survival in its herbivorous niche. For instance, the reduced expression of chemosensory genes may indicate a diminished reliance on olfaction in LDET, favoring a defensive specialization that compensates for the loss of synthetic autonomy for cyanogenic glycosides. Similarly, the downregulation of chitin-binding genes could weaken the epidermal barrier, potentially facilitating venom release while conserving energy for lipid-based protective structures, a shift consistent with the observed emphasis on wax biosynthesis [22,24].

Despite these insights, our study has several limitations that warrant further investigation. First, the absence of a complete genome sequence for *A. yunnanensis* may have hindered the annotation of low-abundance or highly divergent genes critical for cyanogenic glycoside biosynthesis, potentially masking a cryptic biosynthetic pathway that operates at a post-transcriptional or enzymatic level. This limitation underscores the need to investigate whether alternative regulatory mechanisms, such as epigenetic modifications or microRNA-mediated silencing, suppress these genes in LDET under specific environmental cues. Second, our analysis is based solely on transcriptomic data, which does not directly reflect protein abundance or metabolic activity. Future studies should employ LC-MS to directly measure the distribution of cyanogenic glycosides (e.g., linamarin and lotaustralin) in LDET and LP tissues, validating the absence of a biosynthesis signature observed in this study. Integrating proteomics and metabolomics approaches could also provide a more comprehensive view of venom secretion. Finally, our comparison was limited to LDET and LP tissues, and including additional tissues (e.g., hemolymph, fat body) could offer a more holistic understanding of venom synthesis, transport, and storage across the insect body.

## 5. Conclusions

In conclusion, our study reveals a complex, multi-component mechanism in LDET that supports venom secretion through lipid metabolism, redox reactions, and structural adaptations. These processes collectively ensure the stability, activation, and delivery of venom components while protecting the secreting tissue from damage. The resource reallocation strategy observed in LDET underscores the evolutionary trade-offs associated with specialized defense mechanisms in insects. Future research should focus on validating these mechanisms through functional studies, such as RNA interference targeting *FAR* genes to assess their role in venom secretion or gene overexpression to confirm the roles of lipid metabolism and redox reactions in venom function. Additionally, integrating multi-omics data and conducting comparative analyses with other venomous insects will further elucidate the evolutionary and ecological significance of these adaptations, potentially informing novel strategies for pest control.

## Figures and Tables

**Table 1:** Differential expression of cyanogenic glycoside biosynthesis-related genes in LDET and LP tissues of *Achelura yunnanensis* larvae.

Gene_ID	NR Description	Mean TPM (LDET)	Mean TPM (LP)	Log2FC (LDET vs. LP)	*p*-Value	Padj *	Regulation
DN1005_c0_g1	*CYP405A2*, *partial*	213.097	470.398	1.212	0.584	0.732	up
DN3986_c0_g1	*CYP332A3*	53.848	76.196	2.606	0.018	0.060	up
DN3987_c0_g2	*CYP332A3*	69.290	109.101	1.218	0.528	0.688	up
DN7282_c0_g3	*UGT33A1*	1.814	3.568	−0.971	0.954	0.976	down
DN1642_c0_g1	*UGT33A1*	1.551	2.301	1.713	0.237	0.405	up

* adjusted *p*-value, Benjamini–Hochberg correction. None of the genes reached statistical significance (Padj < 0.05).

## Data Availability

The original contributions presented in this study are included in the article/Supplementary Materials. Further inquiries can be directed to the corresponding authors.

## Supplementary Materials

The following supporting information can be downloaded at https://www.mdpi.com/article/10.3390/insects16060588/s1, Table S1: Sequencing Data Summary; Table S2: Optimized Assembly Quality Assessment; Table S3: Principal Component Explanation Table; Table S4: Details of Differential Expression; Table S5: Gene List for Each Region of the Venn Diagram; Table S6: GO Enrichment Analysis of Genes Upregulated in LDET Compared to LP; Table S7: KEGG Pathway Enrichment Analysis of Genes Upregulated in LDET Compared to LP; Table S8: Spearman Correlation and Node Centrality Analysis of Upregulated Interacting Genes in LDET and LP; Table S9: KEGG Pathway Enrichment Analysis of Genes Downregulated in LDET Compared to LP; Table S10: Spearman Correlation and Node Centrality Analysis of Downregulated Interacting Genes in LDET and LP; Table S11: qRT-PCR Validation of Differentially Expressed Genes and Their Primer Sequences.
